# *“Then you are left on your own”*: barriers to guideline-based speech and language therapy for aphasia along the patient journey in the German-speaking healthcare context

**DOI:** 10.1186/s12913-026-15523-w

**Published:** 2026-09-07

**Authors:** Susann May, Felix Muehlensiepen, Laura Plotho, Helmut Ritschl, Robert Darkow

**Affiliations:** 1 German Heart Center of the Charité - Department of Cardiology, Angiology and Intensive Care Medicine, Berlin, Germany; 2 Charité- University Medicine Berlin, Corporate member of Freie Universität Berlin and Humboldt-Universität Berlin, Berlin, Germany; 3 User Experience in Digital Health Lab, Center for Health Services Research, Faculty of Health Sciences, Brandenburg Medical School Theodor Fontane, Rüdersdorf, Germany; 4 Institute for Logopedics, JOANNEUM University of Applied Sciences, Graz, Austria

**Keywords:** Aphasia, Speech and language therapy, Patient journey, Continuity of care, Access to care, Outpatient care, Health services research, Qualitative research, Focus groups, Guideline implementation

## Abstract

**Background:**

Although evidence-based recommendations for aphasia rehabilitation exist, guideline-based speech and language therapy is still not consistently implemented in routine care. Previous studies have described barriers to therapy participation from the perspective of people with aphasia, but less is known about how these barriers develop along the patient journey, especially in outpatient care. This study explored systemic and individual barriers to the uptake of speech and language therapy in aphasia care and identified implications for improving services.

**Methods:**

An exploratory qualitative study was conducted in the German-speaking healthcare context using two online focus group discussions. A multiperspectival sample was recruited, including people with aphasia, relatives, speech and language therapists, and other healthcare stakeholders. Data were collected with a semi-structured topic guide addressing barriers to guideline-based therapy use, challenges along the patient journey, and possible solutions. Audio recordings were transcribed verbatim, anonymized, and analyzed in German using Kuckartz’s structured qualitative content analysis.

**Results:**

The analysis identified six main categories of factors influencing the provision of guideline-recommended speech and language therapy: societal and systemic conditions; access to care; the patient journey and continuity of care; relatives and the social environment; psychosocial factors; and therapy provision and interdisciplinary care; proposed solutions and areas for improvement are reported separately. Findings indicated that the provision of guideline-recommended speech and language therapy is strongly shaped by structural barriers within the healthcare system, particularly in the outpatient sector. Fragmented care pathways, limited coordination, bureaucratic and financial obstacles, and insufficiently accessible or specialized services hindered the implementation of guideline-based care. The transition from inpatient treatment to self-organized outpatient follow-up emerged as a particularly vulnerable stage. Relatives often compensated for deficits in formal care structures by taking on organizational, communicative, and emotional responsibilities. However, this informal compensation also created inequalities, as access to ongoing care partly depended on privately available support. Participants further highlighted shortcomings in interdisciplinary and psychosocial care and emphasized the need for earlier information, structured care pathways, improved coordination, and stronger implementation of evidence-based recommendations.

**Conclusions:**

The provision of guideline-recommended speech and language therapy in aphasia appears to be shaped largely by structural and organizational barriers rather than by individual factors alone. Improving aphasia care will require coordinated action across the healthcare system, especially in outpatient care, including communication-accessible pathways, stronger continuity between sectors, better access to specialized and interdisciplinary services, and less reliance on informal support from patients and families.

**Trial registration:**

This exploratory qualitative study was not prospectively registered.

**Supplementary Information:**

The online version contains supplementary material available at https://doi.org/10.1186/s12913-026-15523-w.

## Background

Aphasia is a common and disabling consequence of stroke. In addition to impairments in speaking, understanding, reading, and writing, it substantially affects communicative participation, social reintegration, quality of life, and mental health. Recent studies have shown that post-stroke aphasia is associated with marked psychosocial burden, including reduced quality of life and a high prevalence of depressive symptoms, underscoring that aphasia is not merely a language disorder but a long-term condition with far-reaching consequences for everyday life and social participation [1], [2].

Speech and language therapy is a core, evidence-based intervention in aphasia rehabilitation. A Cochrane review found that speech and language therapy, compared with no therapy, improves functional communication, reading, writing, and expressive language, while suggesting that greater intensity, higher dose, and longer duration may yield additional benefit for at least some patients [3]. More recently, the European Stroke Organisation guideline on aphasia rehabilitation reinforced the importance of sufficiently intensive and individualized treatment and recommended, among other aspects, a minimum total dose of 20 hours, at least 3 hours of therapy per week, and therapy on at least 4 days per week where feasible and appropriate [4].

This evidence base has become increasingly precise. A landmark randomized controlled trial (FCET2EC) showed that a three-week intensive outpatient programme of 15 hours of individual and group speech and language therapy per week produced significantly greater improvements in everyday verbal communication in patients with chronic aphasia (≥60 months post-stroke) compared with a waiting-list control condition, with effects remaining stable at six-month follow-up [5]. An individual patient data meta-analysis from the RELEASE collaboration, pooling data from 25 randomized controlled trials (*n* = 959), further demonstrated that optimal therapy dosing is not uniform but varies systematically with patient characteristics: younger patients (≥65 years), men, those with mild-to-moderate aphasia, and those in the late subacute or chronic phase responded best to higher treatment intensities (≥9 hours per week), whereas older patients, women, those with moderate-to-severe aphasia, and those in the acute or early subacute phase showed better outcomes at lower intensities (≤2 hours per week) [6–8]. These findings make clear that guideline-based aphasia care is not reducible to a single treatment modality or a uniform dosing regimen, but requires individualized, phase-sensitive, and sustained intervention and that any barrier preventing access to such care carries differentiated consequences depending on the patient’s profile and phase of recovery.

Despite a growing evidence base and increasingly specific guideline recommendations, substantial evidence-practice gaps in poststroke aphasia rehabilitation have been documented internationally [9], and the implementation of guideline-based aphasia care in routine practice remains inconsistent. An updated systematic review of stroke clinical practice guidelines showed that, although many recommendations relevant to aphasia management are available, important gaps remain, particularly with regard to community support, interprofessional practice, and participation-oriented rehabilitation [10]. In the German-speaking healthcare context, available evidence likewise points to a substantial discrepancy between recommended and actual care. A health insurance claims data analysis, for example, reported considerable deficits in adherence to guideline-based speech and language therapy aftercare following stroke [11].

This implementation gap appears to be especially pronounced in the outpatient sector. Whereas care processes in acute stroke care and inpatient rehabilitation are usually more structured, ongoing aphasia care after discharge often depends on medical prescriptions, regional service availability, organizational resources, and private support. In a previous qualitative study, barriers to participation in guideline-based, high-frequency aphasia therapy were initially identified from the perspective of people with aphasia [12]. That study highlighted a range of health system-related, organizational, socioeconomic, disease-related, and patient-related barriers, many of which were located within the healthcare system itself. Reported obstacles included workforce shortages, deficits in professional knowledge, cost-related barriers, prescription-related difficulties, and a lack of patient-centred and family-oriented support [12].

However, identifying barriers is only a first step. Less is known about how these barriers unfold across the patient journey and interact across phases of care. Existing research has often focused on therapy intensity, on the implementation of intensive aphasia services, or on the experiences of specific treatment programmes. For example, Trebilcock and colleagues examined barriers and facilitators to implementing intensive and comprehensive aphasia services across six countries, whereas Babbitt and colleagues explored stakeholder experiences within an intensive comprehensive aphasia programme [13, 14]. Similarly, an Australian experience-based co-design study described unmet care needs across the entire patient journey, with transitions between care settings emerging as particularly challenging [15]. These studies have generated important insights into implementation and service delivery, but they do not fully explain how barriers accumulate across routine care pathways from acute care and rehabilitation to discharge, outpatient care, and longer-term follow-up.

A patient-journey perspective is therefore particularly useful for understanding aphasia care. In health services research, access to care has been conceptualized as a multi-stage process that depends on the interaction between healthcare system characteristics and people’s ability to perceive, seek, reach, pay for, and engage with care [16]. In parallel, continuity of care has been defined as the extent to which care is experienced as coherent and connected over time, especially through informational, management, and relational continuity [17]. Recent work on patient-centred care pathways further emphasizes that care pathways should not be understood merely as technical clinical sequences, but as integrative frameworks that link coordination, information flows, organizational conditions, and patient perspectives across sectors [18]. These concepts are highly relevant to aphasia care in the outpatient setting, where communication impairments, fragmented transitions, workforce shortages, and missing coordination mechanisms may jointly hinder access to and uptake of therapy [15–18].

Against this background, a multiperspectival analysis of barriers along the patient journey of people with aphasia in the German-speaking healthcare context is needed. While the earlier study identified barriers from the perspective of people with aphasia [12], the present study extends this work by contextualizing these findings together with the perspectives of relatives, speech and language therapists, representatives of professional and patient organizations, and other healthcare stakeholders. This broader perspective allows for a more comprehensive examination of how systemic, organizational, individual, and social factors interact across the patient journey, where discontinuities arise, and which practical implications can be derived for improving outpatient aphasia care. The present study therefore aimed to investigate barriers to the uptake of speech and language therapy for aphasia along the patient journey, with a particular focus on outpatient care in the German-speaking healthcare context. Specifically, the study addressed three questions: (1) what factors impact the provision of guideline-recommended speech and language therapy to people with aphasia, (2) which systemic and individual barriers arise along the patient journey, and (3) which practical implications and potential solutions can be derived from these findings?

## Methods

### Study design

We conducted an exploratory qualitative study based on two focus group discussions to examine factors influencing the provision of guideline-recommended speech and language therapy among people with aphasia. Barriers to guideline-based aphasia management have been examined internationally, particularly from the perspective of speech and language therapists [19] and, more recently, of people with aphasia and their supporters [15]. However, multiperspectival evidence on how such barriers unfold along the patient journey in the prescription-based outpatient care systems of Germany and Austria is scarce. An exploratory qualitative focus group design was chosen because it enables interaction between different stakeholder groups, allows perspectives to be contrasted and negotiated within the discussion, and is well suited to generating a system-level understanding of care processes. The data collection was conducted as part of the AWARE project, which aims to identify reasons for the non-receipt of guideline-based treatment, predictors of service utilization, and the needs and preferences of people with aphasia. The study focused on aphasia care in Germany and Austria, with particular attention to care provision in the outpatient setting. The study is reported in accordance with the Consolidated Criteria for Reporting Qualitative Research (COREQ) [20].

### Ethical considerations

This study involved human participants and was approved by the Ethics Committee of the Medical University of Graz (Reference ID: 35–389 ex 22/23). All participants received written information about the purpose and procedures of the study prior to participation. They were informed that participation was voluntary and that they could withdraw at any time without giving reasons. Written informed consent was obtained from all participants before the focus group discussions took place. Personal data were handled in accordance with current data protection standards. Audio recordings and transcripts were pseudonymized, and all findings are reported in anonymized and aggregated form to prevent the identification of individual participants.

### Participants

Participants were recruited using purposive sampling in order to capture a broad range of perspectives on aphasia care. Participants were identified and approached through aphasia self-help groups and patient organizations in Germany and Austria, professional networks of speech and language therapists, and clinical contacts of the project team. Potential participants received written study information and were invited to contact the research team. Recruitment aimed at maximal variation of perspectives rather than at statistical representativeness. The sample was deliberately multiperspectival and included people with aphasia, relatives, speech and language therapists, representatives of professional or patient organizations, some of whom were also personally affected, as well as other stakeholders from the healthcare system. In total, 12 individuals participated in the study, with 6 participants in each focus group. Sample size was guided by the concept of information power: given the focused aim, the multiperspectival and information-rich composition of the groups, and the exploratory design, two focus groups with six participants each were considered sufficient to address the research questions. During analysis, the second focus group yielded predominantly confirmatory content with few new subcategories, suggesting that a satisfactory degree of thematic saturation had been reached for the purposes of this exploratory study. The two focus groups consisted of different participants, but both groups were composed to reflect diverse perspectives on care. Eligible participants were adults (aged 18 years or older) who were either (a) people with aphasia, (b) relatives of people with aphasia, or (c) professionals or other stakeholders with relevant experience in aphasia care. Participants were required to have sufficient German language proficiency to take part in the discussion and to provide informed consent. Individuals were excluded if they were unable to participate meaningfully in an online group discussion or if informed consent could not be obtained. No financial incentives were provided.

### Data collection

Data were collected through two online focus group discussions conducted via videoconference in MS Teams. Each session lasted approximately 90 minutes. Both focus groups were moderated by SM, a speech and language therapist and researcher experienced in aphasia care and qualitative methods, supported by RD and FM as co-moderators, who took field notes and monitored the chat function. Neither moderator had a therapeutic relationship with any of the participants. To support the participation of people with aphasia, the moderators - experienced speech and language therapists in aphasia care - used supported communication strategies, including a slowed pace, simplified and redundant phrasing, closed and yes/no questions where helpful, explicit turn allocation, additional response time, verification of understanding through paraphrasing, and the option to use the chat function. Key questions were provided in advance in plain language, and relatives were able to support participants during the session where needed. A semi-structured topic guide was developed by the interdisciplinary project team, which included expertise from speech and language therapy (R.D. & S.M.), psychology (L.P.), health services research (S.M. & F.M), and social sciences (F.M.). The interview guide was aligned with the study’s guiding research questions and covered three main thematic areas: (1) factors impacting the provision of guideline-recommended speech and language therapy to people with aphasia, (2) systemic and individual barriers along the patient journey, and (3) practical implications and possible approaches to improving care. Prior to data collection, the topic guide was pilot tested and subsequently revised (the topic guide is provided in Supplementary Material 1). In addition to the audio recording of the discussions, field notes were taken to support contextual understanding of the group interactions. However, these field notes were not directly included in the formal analysis. The focus groups were audio-recorded and transcribed verbatim in German. All transcripts were anonymized prior to analysis.

### Data analysis

The data were analyzed using Kuckartz’s structured qualitative content analysis approach [21]. Analysis was supported by MAXQDA Version 26.1.0. A hybrid deductive-inductive coding strategy was applied. In line with Kuckartz’s approach, an initial set of main categories was derived deductively from the study’s research questions and the topic guide. Subcategories were then developed inductively through close reading and iterative coding of the transcripts. During this process, the deductively derived main categories were reviewed against the empirical material and revised where necessary: categories were renamed, merged, or differentiated, and new data-driven categories were added. The final category system was consolidated through regular discussions within the research team. The transcripts were read closely and independently coded by two researchers (S.M. and R.D.), who developed an initial set of categories that was iteratively refined within the team. The analysis was conducted in German. Selected quotations were translated into English for reporting purposes. The research team comprised speech and language therapists, health services researchers, and social scientists with varying degrees of professional involvement in aphasia care. Team members with a clinical background in speech and language therapy brought detailed knowledge of care structures, but also professional assumptions - for example, regarding the value of high-frequency therapy and the deficits of outpatient provision - that may have sensitized the analysis toward system-level barriers. Conversely, team members from health services research and the social sciences approached the material with a stronger focus on organizational and structural explanations. These differing perspectives were made explicit and deliberately used as a corrective during consensus discussions: interpretations were challenged across disciplinary boundaries, negative or deviating cases were actively sought, and the wording of categories was revised where it reflected professional assumptions rather than the participants’ accounts. An audit trail documenting coding decisions and category revisions was maintained throughout the analysis.

## Results

The two focus groups were composed multiperspectivally and brought together participants from different positions within aphasia care, including people with aphasia, relatives, speech and language therapists, and other relevant healthcare stakeholders. Table 1 summarizes the composition of the two focus groups. All three participants with aphasia had acquired aphasia following stroke. At the time of the focus groups, time since stroke ranged from 3 to 15 years, and self-reported aphasia severity ranged from moderate to severe.

**Table 1:** Characteristics and roles of participants in the two focus groups

Focus group participant ID	Country	Participant characteristics
FG1_P1	Austria	Speech and language therapist, inpatient acute care
FG1_P2	Austria	Speech and language therapist, outpatient care
FG1_P3	Austria	Speech and language therapist, inpatient care; head of a self-help group
FG1_P4	Austria	Relative; deputy head of a self-help group
FG1_P5	Austria	Person with aphasia
FG1_P6	Austria	Relative
FG2_P7	Germany	Academic speech and language therapist, outpatient care
FG2_P8	Germany	Person with aphasia; chair of a regional aphasia association
FG2_P9	Germany	Person with aphasia
FG2_P10	Germany	Health services researcher in aphasia care
FG2_P11	Germany	Speech and language therapist, inpatient care
FG2_P12	Germany	Relative

The analysis identified six main categories of factors influencing the provision of guideline-recommended speech and language therapy along the patient journey: (1) societal and systemic conditions; (2) access to care; (3) the patient journey and continuity of care; (4) relatives and the social environment; (5) psychosocial factors; and (6) therapy provision and interdisciplinary care. Proposed solutions and areas for improvement are reported separately.

## Societal and systemic conditions

The analysis showed that structural conditions within the healthcare system had a central influence on the uptake of speech and language therapy. Financial aspects represented a major barrier to access. In particular, the need to pay therapy costs upfront, combined with long reimbursement periods, meant that necessary treatment can only be utilized to a limited extent or not at all. This financial burden directly affected therapy utilization, irrespective of the motivation of those affected.

*Reimbursements take an extremely long time. […] At some point, you simply can’t afford it anymore.* (FG1_P4_relative)

Bureaucratic processes related to the prescription of speech and language therapy were perceived as complex and burdensome. Applying for therapy often required repeated physician contacts and extensive administrative steps. At the same time, those affected reported a sense of having to justify their need for therapy. These processes may lead to delays and constitute an additional challenge, particularly for people with aphasia.

*You are always in the position of a supplicant […]. You always have to explain everything at length.* (FG2_P12_relative)

A further major structural problem was the shortage of speech and language therapists. This resulted in limited availability of therapy slots, long waiting times, and regional disparities in care provision. The shortage affected both general care and specialized services for aphasia. As a consequence, even individuals with clear therapy needs and existing motivation may be unable to receive adequate care.

*I experience staff shortages very strongly. I am a speech therapist in a rural area. […] Yes, there is an enormous shortage of speech therapists.* (FG1_P2_Speech and language therapist)

Regulatory conditions also had a substantial impact on care provision. In particular, the role of physicians in prescribing therapy was described as critical. Uncertainty in dealing with guidelines, as well as fear of economic consequences, meant that necessary prescriptions were not always issued. These regulatory barriers directly affected the availability of therapy and intensify existing problems in care provision.

*Yes, it is exhausting to obtain a prescription. Sometimes doctors do not prescribe it either.* (FG1_P2_Speech and language therapist)

At the same time, participants described aphasia as a condition with limited societal visibility. The condition was perceived as little known, both in the general public and in parts of the healthcare system. This limited awareness contributed to misunderstandings in everyday life and to the insufficient recognition of the needs of people with aphasia.

*Aphasia? What is aphasia? First you have to inform yourself about it, usually via Google.* (FG1_P6_relative)

A central problem also lay in the limited visibility of people with aphasia in public space. Many withdrew from social life or were not reached by support services, which hindered both social integration and access to support.

*And then of course, if you have a partner, it is much easier for a person with aphasia. People who live alone have it very hard. They are isolated and are not reached at all by support services.* (FG1_P6_relative)

## Access to care

In both focus groups, a central starting point for improving care was identified in the provision of information. Early and comprehensible information was seen as essential for enabling people with aphasia and their relatives to orient themselves within the healthcare system and identify suitable services. The provision of information was described as a basic prerequisite for adequate care.

*Yes, I think we need to start very early in giving patients and relatives information. Lists of speech therapists, for example, or information on where they can find out more about support options or self-help groups. I think this has to happen very early …* (FG1_P3_Speech and language therapist)

At the same time, the analysis showed that such information was often lacking in the acute phase. This concerned both the general understanding of aphasia and knowledge of available therapy options and points of contact.

*In the acute phase, it often becomes apparent that aphasia and speech therapy as a whole are very unfamiliar to both patients and relatives. Many are surprised that language can also be affected.* (FG1_P1_Speech and language therapist)

The lack of information thus constituted an early barrier within the patient journey and contributed to difficulties in navigating subsequent care.

Beyond the lack of early information described above, access to speech and language therapy was shaped by further practical and individual conditions. Organizing therapy appointments constituted a particular challenge for people with aphasia. Language impairments made independent communication with practices and institutions more difficult. This increased dependence on support from others and may result in delays or even complete non-use of therapy.

*It is often difficult for people with aphasia to get to therapy, yes. Just making therapy appointments is difficult because of the language barrier.* (FG1_P3_Speech and language therapist)

Mobility restrictions represented a further barrier. In addition to physical impairments, insecurity in the use of public transport and communicative barriers also played a role. These particularly hindered access to outpatient services. Restricted mobility may lead to situations in which available services cannot be utilized.

*It is very, very difficult even to explain to a taxi driver where you want to go. Yes, the whole thing becomes quite cumbersome for people with aphasia.* (FG1_P3_Speech and language therapist)

Alongside individual and organizational barriers, participants also described a lack of suitable care services. This concerned both the number of available therapy slots and the extent to which available services matched the needs of those affected. Inadequate service structures therefore meant that therapy may not be utilized despite existing motivation.

*It is extremely difficult even to find speech therapists for aphasia, or […] to find any who actually have free slots.* (FG1_P6_relative)

## Patient journey and continuity of care

The analysis showed that the care of people with aphasia was characterized by marked discontinuities over time. A central problem concerned the break between inpatient and outpatient care. After discharge from acute care or rehabilitation, structured continuation of therapy was often lacking. As a consequence, those affected may experience periods without any care at all. Transitions within the healthcare system were described as uncoordinated and strongly dependent on chance.

*In Germany, we have no follow-up care for people with aphasia. You can get into the stroke unit very well, you get into […] very quickly, but then you are out there on the market, so to speak, alone, and you do not know where to go. You simply do not know.* (FG2_P8_ Person with aphasia)

Long-term care for people with aphasia was described as insufficient. After the completion of intensive treatment in the acute phase or rehabilitation, sustainable follow-up structures were lacking. Care was often not oriented toward the chronic nature of the condition. As a result, those affected were left to manage on their own over the longer term.

*Then nobody talks anymore about the long-term consequences after successfully surviving a stroke. That means everything worked as it should, and then you leave with the damage and are left standing there on your own.* (FG2_P9_ Person with aphasia)

Another central aspect was the absence of clear care structures and coordinating entities. There were no standardized care pathways that govern transitions between the different sectors of care. As a result, those affected were strongly dependent on individual initiative or external support. Care was therefore experienced as unsystematic and highly dependent on chance.

## Relatives and the social environment

The analysis highlighted the central importance of relatives and the social environment for the care of people with aphasia. Relatives played a decisive role in organizing and facilitating therapy. They took on tasks such as arranging appointments, gathering information, and accompanying individuals to therapy. In addition, they contributed to motivation and emotional support. People without a supportive social environment were clearly disadvantaged. Without help with organization and communication, it became more difficult for them to use therapy services. This resulted in unequal care situations. Individuals living alone in particular were more frequently affected by underprovision.

*He has to go there, and then we have to do this, and then we have to do that. We do a lot together at home; we also practice writing […]. But he needs my help.* (FG1_P6_relative)

## Psychosocial factors

In addition to structural and organizational aspects, psychosocial factors played a central role in the care of people with aphasia. Many participants reported loneliness and social isolation. Communication difficulties hindered participation in social life and often led to withdrawal. This may negatively affect motivation for therapy. Isolation was described as a central problem by both people with aphasia and their relatives.

*If you have a partner, it is much easier for a person with aphasia. People who live alone, I imagine, have a very hard time.* (FG1_P6_relative)

Those affected often experienced insecurity in social interactions as well as fear of negative judgment. Communication difficulties caused them to hold back in everyday life or avoid social situations. This reinforced withdrawal and made active participation in society more difficult.

*Aphasia does something to people. It is not just a language deficit; people are afraid of being seen as stupid, and I think that leads them into depression, into insecurity, yes, so that they no longer dare to go out.* (FG1_P3_Speech and language therapist)

Motivation for therapy was closely linked to individual experiences and psychosocial factors. While some individuals actively engaged in rehabilitation, others showed more resigned tendencies. Negative experiences, lack of progress, or structural barriers may lead to declining motivation. Attitudes toward therapy thus substantially influenced its utilization.

*They just give up more quickly, become frustrated more quickly, and say it does not work anyway, it does not matter.* (FG1_P1_Speech and language therapist)

## Therapy provision and interdisciplinary care

The analysis showed that, in addition to access to therapy, the design and quality of therapy also played an important role. Adapting therapy to individual needs was described as a central factor for success. Different levels of severity, goals, and life situations required flexible and patient-centered approaches. Standardized care often failed to meet these individual requirements.

*I see it as my task as a therapist to always adapt my therapy to the patient, to their personality, of course also to their wishes […] I think success and whether therapy is accepted depend on that.* (FG1_P2_Speech and language therapist)

Group therapy was described as a valuable complement to individual therapy. It enabled social interaction, mutual support, and communication practice in more everyday contexts. At the same time, structural and organizational barriers to its implementation were reported.

*I see great advantages if group therapy could be offered, for example […]. As for group therapy services, I have not found any anywhere so far.* (FG1_P4_relative)

Home visits were considered an important measure for improving access to therapy, especially for people with mobility limitations. At the same time, structural and organizational restrictions were also evident in this area.

*Until a year ago, it was basically a charitable act on our part when we did home visits […] if I do not do it, nobody does.* (FG1_P2_Speech and language therapist)

The quality of care was also influenced by the degree of specialization of therapists. Participants reported that not all speech and language therapists had sufficient experience in the field of aphasia, which may impair the effectiveness of therapy.

*The field of child language [training opportunities] is enormously well developed. In a year, I can choose from 300 training courses in child language, but maybe only 10 in aphasia and apraxia of speech. (FG2_P11_Speech and language therapist)*

The analysis showed that the care of people with aphasia does not consist exclusively of speech and language therapy, but requires an interdisciplinary approach. In addition to language therapy, neuropsychological and psychosocial support services in particular were described as relevant. At the same time, clear deficits in interdisciplinary collaboration and in access to such services were evident (see also Psychosocial factors).

## Access to psychological and neuropsychological support

In addition to language impairments, cognitive and emotional changes often occurred and required additional therapeutic support. The integration of neuropsychological services was therefore described as an important component of care. At the same time, such services were not available across the board.

*We could not find a psychologist or psychotherapist, and it was not approved either, because the health insurance fund said: if someone cannot speak, then they also cannot talk things through with a psychologist.* (FG2_P12_relative)

Access to psychotherapeutic services was described as severely restricted. In particular, communication impairments meant that those affected were excluded from such services or did not obtain approval for them. As a result, psychosocial burdens were not treated adequately.

*I think psychotherapists find it very, very difficult to work with a person with aphasia […] they need experience with people with aphasia, and you simply do not find that.* (FG1_P3_ Speech and language therapist)

**Fig. 1:**
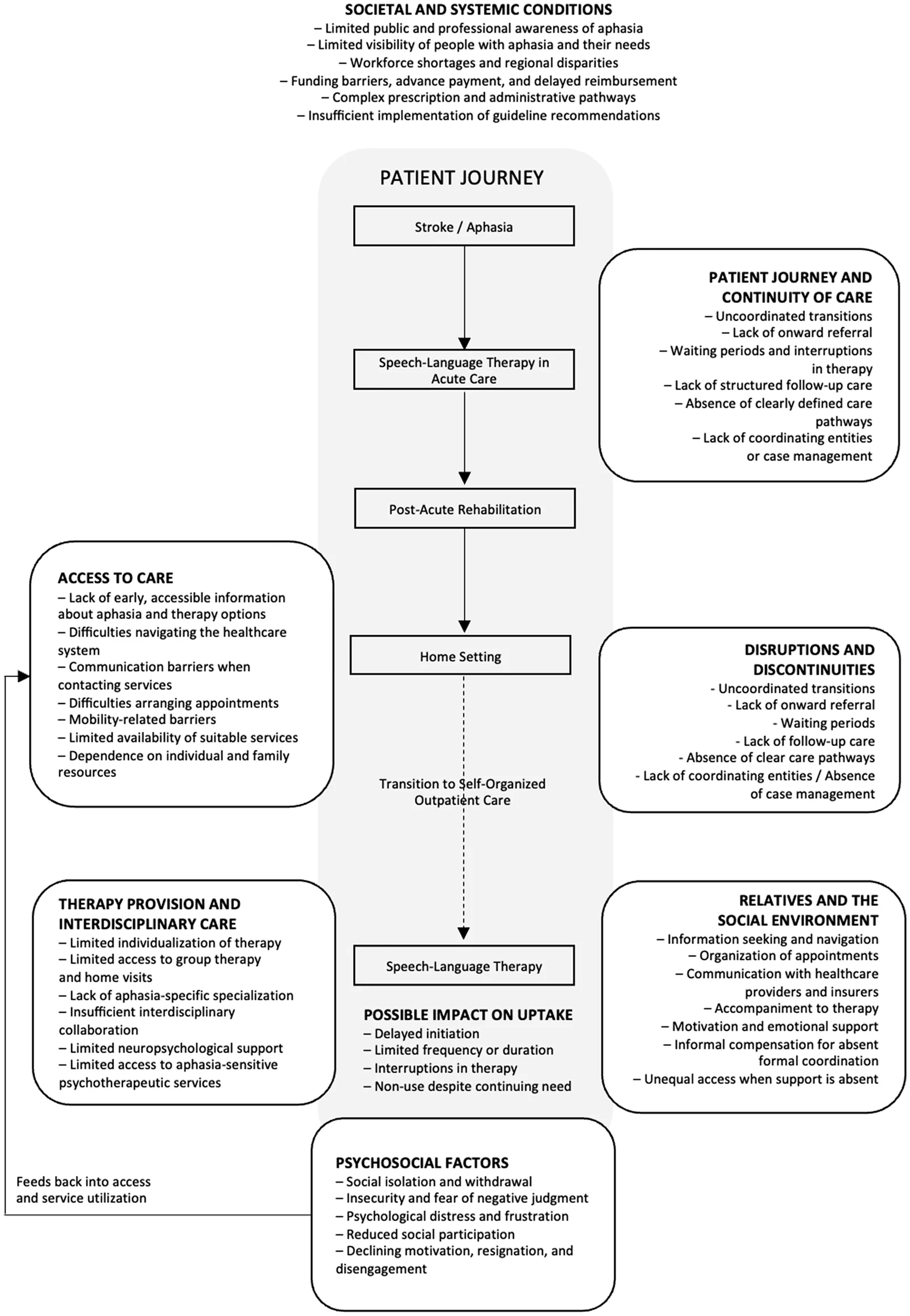
Conceptual representation of factors influencing the provision of guideline-recommended speech and language therapy along the patient journey of people with aphasia (coding tree)

Based on the qualitative content analysis, a conceptual representation of the care situation of people with aphasia was developed (please see Fig. 1). It provides an analytical condensation of the reconstructed barriers, influencing factors, gaps in care, and the relationships between them.

## Solutions and areas for improvement

Both focus groups formulated a wide range of proposals for improving care. A central starting point concerned improving the information available. Early and comprehensible information was intended to help people with aphasia and their relatives orient themselves within the healthcare system and identify suitable services. The provision of information was regarded as a basic prerequisite for adequate care. A frequently mentioned proposal is the development of central platforms or registers that bundle information about therapy services. These were intended to create transparency and facilitate access to appropriate services. The lack of structured information systems was described as a major deficit.

*We would need navigators, or databases, or lists of providers. Who offers what*? (FG2_ P7_Speech and language therapist)

Another central point is the development of clear care pathways. These were intended to improve continuity of care and structure transitions between different sectors of care. Coordinating functions, such as case management, were described as helpful.

*… what we see in oncology is a care pathway that includes specific outcome assessments at very specific time points. We do not have that in stroke aftercare … and we do not have that in aphasia care either.* (FG2_P11_Speech and language therapist)

The importance of scientific evidence was highlighted particularly in the second focus group. Evidence-based guidelines were expected to contribute to the standardization of care and to improve prescribing practices. At the same time, it became clear that existing evidence is not always adequately implemented.

*… these groups of physicians essentially need to have documents in their hands that they can consult quickly. What is it about? What is the first, second, third step? What is the hierarchy of interventions? What is most important, what are the recommendations regarding frequency? That has to be written down somewhere. Ideally, it should be in the guidelines. The guidelines are enormously important, enormously important.* (FG2_P9_Person with aphasia)

The further development of speech and language therapy services was also linked to the qualifications of professionals. In particular, further training and specialization in the field of aphasia were described as necessary.

*… we need good training opportunities, and I am speaking from the perspective of someone who actually treats aphasia and needs regular further training …* (FG2_P11_ Speech and language therapist)

In addition to service-level measures, political strategies were also considered necessary. These included, for example, adapting regulations, improving financing, and strengthening advocacy, while the improvement of care was understood as a broader societal responsibility.

*… I would really like to see more support from the state … for example, saying that aphasia associations actually need a managing director who is not aphasic but can communicate with people with aphasia …* (FG2_P12_relative)

## Discussion

This study shows that the limited uptake of speech and language therapy among people with aphasia is best understood not only as an individual failure to engage with treatment, but as the result of multiple interacting barriers along the patient journey. Across both focus groups, participants described a fragmented pattern of care in which structural constraints, limited access to outpatient services, discontinuities between sectors, and insufficient coordination jointly hindered the implementation of guideline-based aphasia therapy. Taken together, the findings suggest that the reduced uptake of aphasia therapy in the German-speaking healthcare context appears to be rooted less in lack of motivation than in a fragmented outpatient care system that places substantial navigational, organizational, and communicative demands on those affected.

A central finding of this study is that the reduced provision of speech and language therapy in aphasia should be interpreted primarily as a structural rather than an individual problem. Although motivation and personal circumstances were discussed by participants, the focus group data suggest that reduced uptake is largely shaped by barriers within the healthcare system itself, including financing arrangements, prescription procedures, limited service availability, and a lack of transparent information about where and how to access care. This interpretation is consistent with our previous qualitative study, in which people with aphasia described non-participation in guideline-based therapy as closely tied to health system factors, organizational constraints, and socioeconomic barriers rather than to simple unwillingness to engage in treatment [12]. This reading is also supported by the broader health services literature. Levesque and colleagues conceptualize access to care as a multi-stage process that depends not only on individual help-seeking, but also on the visibility, availability, affordability, and appropriateness of services. From this perspective, underuse cannot be reduced to patient preference or adherence; instead, it reflects the interaction between system characteristics and people’s ability to perceive, seek, reach, pay for, and engage with care [16]. In our data, several of these dimensions were affected simultaneously: participants reported poor service visibility and orientation, difficulties obtaining prescriptions, financial strain due to upfront payment and delayed reimbursement, and restricted access to suitable providers. These findings suggest that barriers accumulate across multiple points of the care process rather than operating in isolation, similar to findings in post-stroke aphasia care in other countries such as Australia [15].

This structural interpretation is directly supported by evidence demonstrating that effective intensive aphasia programmes exist and are feasible. The FCET2EC trial, for example, showed that a three-week intensive outpatient programme produced durable improvements in everyday verbal communication in patients with chronic aphasia improvements that persisted over at least six months [5]. Such programmes are, in principle, implementable within existing clinical structures: the FCET2EC protocol was delivered in standard outpatient settings and did not require specialist infrastructure beyond adequate staffing. Similarly, the Big CACTUS trial demonstrated that even one hour per week of computer-based word-finding practice as an add-on to usual care produced significant benefits over six months compared to usual care alone [22]. The fact that such evidence-based options remain largely unavailable in routine German-speaking outpatient care, as suggested by our data, points to a structural implementation failure rather than a limitation of the evidence base itself.

Another key finding of this study is that the most consequential break in aphasia care does not appear to occur during acute treatment, but rather at the transition from inpatient care and rehabilitation to self-organized outpatient follow-up. Participants described acute care and rehabilitation as comparatively structured phases, whereas after discharge the responsibility for initiating and maintaining further therapy was largely shifted to patients and their families. In this sense, the outpatient sector emerged as the point at which guideline-based care became most vulnerable to disruption. This finding is consistent with concepts of continuity of care. Haggerty and colleagues describe continuity as the extent to which care is experienced as coherent and connected over time, particularly through informational, management, and relational continuity [17]. In our data, all three dimensions appeared compromised in the outpatient phase: information about follow-up options was often lacking, transitions between sectors were poorly coordinated, and stable coordinating relationships were largely absent. The resulting care trajectories were experienced as fragmented, unsystematic, and dependent on chance rather than on structured pathways. Our findings also align with broader work on patient-centred care pathways. Care pathways are increasingly understood not merely as technical clinical sequences, but as integrative frameworks that organize care across settings and over time, linking coordination, information flows, and patient-centred decision-making [18]. From this perspective, the absence of structured aphasia pathways in the outpatient sector is not simply an organizational inconvenience; it represents a fundamental weakness in service design. In particular, the lack of clearly defined follow-up responsibilities, referral mechanisms, and coordinating functions such as case management appears to undermine continuity precisely at the point where ongoing therapy would be most needed. The outpatient setting is particularly critical because aphasia often requires sustained, longer-term rehabilitation beyond the acute phase [4]. Our findings suggest, however, that care systems are insufficiently geared toward this longer-term trajectory. Once intensive treatment ends, patients may face waiting times, unclear responsibilities, and the need to organize follow-up care on their own. This is consistent with previous findings from our earlier qualitative study, which showed that non-participation in guideline-based aphasia therapy is strongly shaped by system-level barriers in the post-acute phase [12]. Comparable discontinuities at the transition from acute care have been described in the Australian context, where people with aphasia and their supporters likewise reported unmet needs concentrated at points of transition between care settings [15] - suggesting that, while the specific regulatory mechanisms differ, transition-related vulnerability is a cross-system phenomenon. Notably, the improvement priorities articulated by our participants - early and accessible information, structured provision of therapy, and clearer care pathways - closely mirror the implementation priorities recently established through end-user involvement for post-stroke aphasia services (assessment, aphasia-friendly information provision, and provision of therapy) [23], suggesting considerable international convergence on what should be improved first. Together, these findings suggest that underuse in aphasia care is not only a matter of access, but also of continuity.

The consequences of this structural gap are further amplified by the finding that guideline-based aphasia care requires individualized dosing rather than a one-size-fits-all approach. The RELEASE collaboration showed that the optimal therapy intensity varies substantially by patient profile and rehabilitation phase [6–8]. When patients cannot access any sustained therapy in the outpatient phase, they are deprived not merely of a generic intervention, but of an individualized treatment plan that would differ meaningfully depending on their age, sex, aphasia severity, and time since stroke. The structural barriers identified in our data may therefore not only reduce the amount of therapy provided, but may also make it more difficult to deliver the sustained and individually tailored care supported by current evidence. In this sense, the implementation gap documented here is not only a matter of access in general, but of access to care that is appropriately dosed, timed, and tailored.

Relatives appear to play a role in aphasia care that extends well beyond accompaniment. Rather, they assume key organizational, communicative, and emotional responsibilities and thus effectively function as informal coordinators of care. In doing so, they compensate for deficits in a healthcare system in which formal navigational and coordinating structures are often lacking. This interpretation is consistent with aphasia-specific evidence showing that caregivers adopt multiple roles, such as advocate, motivator, and guardian, in order to fill service gaps and facilitate access to outpatient rehabilitation [24]. At the same time, this informal compensation may generate new inequalities. People with aphasia who have a supportive social environment are better positioned to organize and sustain their care, whereas those who live alone or are less socially connected may be at particular risk of underprovision. More broadly, the literature suggests that caregivers’ ability to access and use formal services is itself shaped by barriers such as costs, poor service quality, and deficient information about available supports, which may further reinforce unequal care trajectories [25].

A particularly important finding of this study is that aphasia itself may become a barrier to accessing aphasia care. Participants described how communication impairments complicate key steps in the care process, including making appointments, explaining their needs, navigating referral pathways, and interacting with healthcare providers and institutions. In this sense, the very condition for which therapy is needed may simultaneously hinder access to that therapy. This interpretation is consistent with Levesque’s model of access, which defines access not only in terms of service availability, but also in relation to people’s ability to perceive care needs, seek help, reach services, and engage with care [16]. Recent qualitative research on transitions in stroke care has shown that communication difficulties in people with aphasia can lead to substantial mismatches between care needs and the care actually received, particularly at transitional points in the post-acute pathway [26]. Similarly, a systematic qualitative review of communication challenges in chronic aphasia found that people with aphasia experience persistent barriers in accessing local services and that individual coping strategies alone are insufficient unless the communicative accessibility of care is improved at a structural level [27]. These findings closely align with our data, in which standard healthcare processes appeared to presuppose a level of linguistic and organizational competence that may be particularly difficult for people with aphasia to maintain. From a health services perspective, this points to a form of communicative exclusion embedded in routine care processes. If healthcare systems rely heavily on verbal negotiation, self-advocacy, and independent navigation, people with aphasia are structurally disadvantaged, even where services formally exist. This suggests that improving aphasia care requires more than simply expanding therapy capacity. Addressing this form of communicative exclusion is not solely a task for speech and language therapy: training all healthcare workers to support communication with people with aphasia is itself a guideline recommendation [10], and its implementation would directly reduce the access barriers described here.

Aphasia was not experienced as an isolated language disorder, but as a condition frequently accompanied by cognitive, emotional, and psychosocial challenges. Accordingly, participants emphasized the relevance of neuropsychological and psychotherapeutic support, while at the same time describing substantial barriers to accessing these services. This suggests that aphasia care should not be understood as an exclusively speech and language therapy issue, but rather as an interdisciplinary care task. Previous research suggests that psychological and psychotherapeutic support for people with aphasia is often insufficiently integrated into routine care. Speech and language therapists have reported substantial barriers to addressing psychosocial well-being and to referring patients on to mental health professionals, including limited service availability, insufficient expertise in aphasia, and poor accessibility of mental health services [28]. More broadly, work on living well with aphasia has shown that access to relevant supports, including mental health services, is often inconsistent [29].

### Practical implications

Based on the findings, several practical implications can be derived for improving aphasia care (please see Table 2).

**Table 2:** Practical implications for improving aphasia care derived from the qualitative findings

Finding	Care-related problem	Practical implication
Lack of information about aphasia, therapy options, and available points of contact	People with aphasia and their relatives have difficulty orienting themselves within the healthcare system; access to therapy is already impeded at an early stage	Early, comprehensible, and standardized information during acute care and rehabilitation; accessible informational materials; systematic signposting to outpatient services and self-help resources
Insufficient orientation at the transition to outpatient care	Care often breaks down after discharge or becomes dependent on chance	Structured discharge and transfer procedures; clearly defined responsibilities for follow-up care
Lack of coordinating structures / absence of case management	Patients and relatives must organize care largely on their own, increasing the risk of underprovision	Introduction of navigator or case management functions; coordinating roles between acute care, rehabilitation, and outpatient services
Bureaucratic and complex prescription procedures	Access to therapy is delayed; communicative demands create an additional burden for people with aphasia	Simplified prescription and application procedures; clearer and more communication-accessible administrative processes
Upfront payment and long reimbursement periods	Financial strain limits the actual uptake of therapy	Transparent information on costs; direct billing arrangements wherever possible instead of advance payment
Workforce shortages and regional disparities	Therapy slots are scarce, waiting times increase, and specialized care is unevenly distributed across regions	Expansion of outpatient capacities; stronger regional networking; consideration of digital and hybrid service models
Lack of specialization in aphasia care	Available therapy is not always needs-based; quality and effectiveness may be compromised	Expansion of aphasia-specific training and continuing education; support for specialized practices and competence centers
Limited access to group-based services and home visits	Important complementary therapy formats are difficult to access in routine care	Development of group-based and outreach services; promotion of flexible service formats
Lack of neuropsychological and psychotherapeutic services	Cognitive, emotional, and psychosocial needs often remain insufficiently addressed	Stronger interdisciplinary collaboration; low-threshold access to psychological and psychotherapeutic support
Relatives assume key coordinating functions	Care is informally compensated; people without a supportive social environment are disadvantaged	Systematic involvement, information, and support for relatives; development of supportive accompanying structures
Communication impairment itself hinders access to therapy	Standard healthcare processes presuppose communicative competence and structurally disadvantage people with aphasia	Communication-accessible contact pathways; assisted appointment scheduling; aphasia-sensitive communication in healthcare institutions
Low awareness and visibility of aphasia	Support needs are insufficiently recognized socially and institutionally	Public awareness activities; greater visibility of aphasia in information and service structures
Lack of structured care pathways	Care remains fragmented and poorly predictable	Development of standardized care pathways for aphasia; clearly defined handover points
Insufficient implementation of evidence-based guidelines	Guideline recommendations are not consistently translated into routine care	Practice-oriented guideline materials; training for prescribing professionals; implementation strategies
Underprovision reinforces psychosocial burden and social withdrawal	The consequences of inadequate care negatively affect therapy use and participation	Early psychosocial support; active follow-up of patients at risk

### Strength and limitations

This study has several strengths. Its multiperspectival qualitative design, which included people with aphasia, relatives, speech and language therapists, and other healthcare stakeholders, enabled a broader understanding of barriers along the patient journey than would have been possible from a single perspective alone. The focus on outpatient care in the German-speaking healthcare context allowed for a detailed examination of a phase of care that appears particularly vulnerable to discontinuities. In addition, the analysis was based on two focus groups with different participant constellations and was conducted independently by two researchers using a structured qualitative content analysis approach, supporting methodological rigor and analytical transparency. Several limitations should also be acknowledged. The study was based on a relatively small qualitative sample and was not intended to generate statistically generalizable findings. Only three participants with aphasia took part, compared with a larger number of professionals and relatives. Their perspectives may therefore be underrepresented relative to those speaking about, rather than from, the experience of aphasia - although one participating relative and one professional also held roles in aphasia self-help organizations and thus contributed additional lived-experience proximity. Moreover, the online focus group format, while lowering geographical barriers, presupposes a degree of verbal fluency, technical competence, and stamina that may have discouraged or excluded people with more severe aphasia. Consequently, the views of people with severe aphasia - who arguably face the most substantial access barriers - are likely underrepresented, and the barriers reported here may underestimate the difficulties experienced by this group. Future studies should employ aphasia-friendly, supported data collection formats (e.g., individual interviews with supported communication techniques, pictorial materials, or proxy-assisted formats) to include this population more fully. The multiperspectival group composition may also have influenced the discussion dynamics, as different stakeholder groups may vary in how openly they express their views in a shared setting. Finally, the findings relate to the statutory-insurance-based, prescription-dependent outpatient care systems of Germany and Austria and cannot be directly transferred to other healthcare contexts. As with qualitative research more generally, the findings reflect subjective experiences and interpretations and should therefore be understood as an exploratory contribution to a field that has so far received limited systematic attention.

## Conclusion

This study indicates that the provision of guideline-recommended speech and language therapy in aphasia is strongly shaped by structural barriers within the healthcare system, particularly in the outpatient sector. Fragmented care pathways, limited coordination, bureaucratic and financial obstacles, and insufficiently accessible and specialized services appear to undermine the implementation of guideline-based care across the patient journey. While individual motivational factors may also play a role, our findings suggest that current care structures place substantial and often unreasonable navigational, organizational, and communicative demands on people with aphasia and their families. Improving aphasia care therefore requires more than expanding therapy capacity alone. It calls for structured outpatient pathways, earlier and more accessible information, stronger coordination across sectors, and better access to specialized and interdisciplinary support. Meaningful improvement in aphasia care will require system-level reform rather than continued reliance on patients and families to compensate for structural deficits.

## Electronic supplementary material

Below is the link to the electronic supplementary material.

Supplementary material 1

## Data Availability

Data are available upon reasonable request. All data relevant to the study are included in the article or uploaded as supplementary information. For further questions regarding the reuse of data, please contact the corresponding author (https://felix.muehlensiepen@mhb-fontane.de).

## Declarations

### Patient and public involvement

Patients and/or the public were not involved in the design, or conduct, or reporting, or dissemination plans of this research. People with aphasia and their relatives participated as research participants in the focus group discussions; this is distinct from formal patient and public involvement in study design, analysis, or reporting.
