# Supplementary material for: “Then you are left on your own”: barriers to guideline-based speech and language therapy for aphasia along the patient journey in the German-speaking healthcare context
Source: BMC Health Serv Res. 2026 Sep 7;26:1239. doi: 10.1186/s12913-026-15523-w (PMC13551913; doi:10.1186/s12913-026-15523-w)
Supplement: Supplementary file 1 [file 12913_2026_15523_MOESM1_ESM.pdf]

Focus group interview guide

**Guiding question / narrative prompt**	**Check aspects**	**More specific questions**	**Follow-up and steering questions**
Could you briefly introduce yourself and tell us in what role you are here today?	Warm-up: introduction		
**Input:** In our results, we see that people with aphasia do not make use of as much speech and language therapy as recommended by the guidelines. We have observed that the reasons preventing access are often systemic in nature (costs, lack of prescriptions, shortage of speech and language therapists, effort involved in obtaining prescriptions).	Validation of the quantitative results / generation of interpretive approaches	To what extent do the presented results correspond with your own experiences?<br><br>Do the results surprise you?<br><br>Have you had similar experiences / observations?<br><br>Where do you see deviations or discrepancies?<br><br>What can we do to ensure that finding a speech and language therapist is not simply a matter of luck?<br><br>In the previous focus group, we heard that the social environment (family and friends) plays a key role in the uptake of speech and language therapy, and that people fall through the cracks when this support is lacking. What can we do to counteract this?<br><br>What measures could help overcome these barriers?	Could you tell us more about that?<br><br>And then what happened?<br><br>How was that for you?<br><br>How do you see that?<br><br>Could you elaborate on that?<br><br>Could you give an example?<br><br>What exactly do you mean by that?
How can we ensure that all people with aphasia are equally considered in therapy? What would need to change so that you, or people with aphasia, make use	Derivation of practical implications	What additional support services would help?<br><br>How could speech and language therapy practices better respond to these needs?<br><br>What measures could be taken to make access to speech and language therapy easier?	Could you tell us more about that?<br><br>And then what happened?<br><br>How was that for you?

of therapy more often?		In your opinion, what health policy measures would be necessary to improve speech and language therapy provision for people with aphasia?<br><br>What changes in the regulatory framework would be necessary to facilitate and promote access to speech and language therapy?<br><br>What support is needed from payers, health insurance funds, or social insurance providers?<br><br>What do you observe with regard to the continuity of speech and language therapy care?<br><br>What disruptions in care are observed?<br><br>At the individual level, care depends on the social environment or also on whether someone lives in an urban or rural area. Wouldn't a care pathway, or a systematic, standardized, and quality-assured care procedure, make sense here? In oncology, this works well. Why does it not succeed in aphasia care?<br><br>Could one imagine working toward this?<br><br>Against the background of your own experiences: what are resources / starting points, or also challenges, for ensuring systematic, standardized, and quality-assured care?<br><br>What strategies could help ensure the continuity of speech and language therapy care, especially after discharge from hospital or	How do you see that?<br><br>Could you elaborate on that?<br><br>Could you give an example?<br><br>What exactly do you mean by that?

		after the completion of a therapy phase?	
